# Trigger zones in trigeminal neuralgia: clinical features, pathophysiological mechanisms, and therapeutic strategies

**DOI:** 10.3389/fneur.2026.1838454

**Published:** 2026-07-31

**Authors:** Xiaoyan Li, Huilin Liu

**Affiliations:** Beijing Key Laboratory of Acupuncture Neuromodulation, Beijing Hospital of Traditional Chinese Medicine, Capital Medical University, Beijing, China

**Keywords:** central sensitization, peripheral sensitization, targeted therapy, trigeminal neuralgia, trigger zone, voltage-gated sodium channels

## Abstract

Trigeminal neuralgia (TN) is a neuropathic pain disorder characterized by recurrent, unilateral, electric shock-like facial pain, with trigger zones representing one of its most distinctive clinical features. These localized hypersensitive regions can be reliably activated by innocuous mechanical stimuli such as light touch, speech, or chewing, and their distribution provides important guidance for localizing the affected trigeminal branch and directing treatment decisions. Current evidence suggests that trigger zone formation involves peripheral nerve hyperexcitability driven by upregulation of voltage-gated sodium channels, mechanical sensitization from adjacent facial and masticatory muscle activity, and central sensitization within the trigeminal brainstem complex. At the brainstem level, abnormal activation of wide dynamic range neurons results in the misinterpretation of innocuous tactile input as pain. Clinically, systemic sodium channel blockers remain the foundation of initial treatment, while trigger zone-targeted interventions including peripheral nerve blockade, botulinum toxin type A injections, and minimally invasive ablative techniques have emerged as important adjunctive approaches. The absence of standardized objective assessment criteria and insufficient comparative clinical evidence remain key limitations in the field. A more thorough understanding of trigger zone pathophysiology may ultimately support the development of precision-based, individualized management strategies for TN.

## 1 Introduction

Trigeminal neuralgia (TN) is a neuropathic pain disorder characterized by recurrent, unilateral, electric shock-like facial pain of extreme severity (1). Individual attacks typically last from a fraction of a second to 2 minutes and may recur dozens to hundreds of times per day during active phases. Population-based studies estimate an annual incidence of 4 to 13 per 100,000 individuals, with markedly higher rates observed beyond the sixth decade of life (2, 3). A consistent female predominance has been reported across multiple populations, with female-to-male ratios ranging from approximately 1.5 to 2.1. Due to the sudden onset and overwhelming intensity of attacks, many patients develop pronounced avoidance behaviors affecting eating, speaking, and facial hygiene. These changes result in substantial functional impairment and psychosocial burden.

According to the International Classification of Headache Disorders third edition (ICHD-3), TN is classified into classical, secondary, and idiopathic subtypes. Classical TN, which accounts for approximately 75% of cases, arises from neurovascular compression at the trigeminal root entry zone, most commonly involving the superior cerebellar artery (4). Persistent pulsatile compression at this site induces focal demyelination at the Obersteiner-Redlich zone, where the transition from central to peripheral myelin renders axons particularly vulnerable to mechanical injury. Demyelinated fibers develop abnormal membrane excitability, facilitating ectopic impulse generation and ephaptic cross-excitation between adjacent tactile and nociceptive afferents (5). This cascade provides a mechanistic basis for the cardinal clinical features of TN, including paroxysmal attacks, post-paroxysm refractory periods, and the characteristic responsiveness to sodium channel blockers. Notably, neurovascular contact alone is observed in a meaningful proportion of asymptomatic individuals, and only compression producing demonstrable morphological nerve changes reliably distinguishes symptomatic from asymptomatic sides (6). This distinction has become central to contemporary diagnostic grading systems, which require radiological evidence of nerve displacement, distortion, or atrophy at the compression site to confidently diagnose classical TN, rather than contact alone. The mechanistic and trigger zone evidence reviewed in this article is drawn predominantly from classical and idiopathic TN; secondary TN, particularly cases associated with multiple sclerosis, falls outside the primary scope of this review (7).

Among the defining clinical features of TN, trigger zones represent one of the most characteristic and diagnostically informative findings (8). These are localized hypersensitive regions on the face or within the oral cavity where normally innocuous mechanical stimuli, including light touch, speech, chewing, or tooth brushing, can reliably precipitate paroxysmal pain attacks (9). Trigger zones are identifiable across a broad spectrum of 36 to 97% of TN patients within published cohorts; this wide prevalence band is primarily driven by inter-study disparities in bedside assessment workflows, diagnostic thresholds for valid trigger zone definition, and patient recruitment frameworks, rather than inherent biological divergence among TN populations (10, 11). Their anatomical distribution closely follows the topography of the affected trigeminal branch, with the perioral and nasal regions most frequently involved. Beyond their diagnostic value, trigger zones contribute substantially to avoidance behavior and social withdrawal, as patients consciously minimize any stimulation of sensitive facial areas (12). Despite their well-recognized clinical importance, the pathophysiological mechanisms underlying trigger zone formation remain incompletely understood, and no standardized protocol currently exists for their objective assessment.

Current management of TN relies primarily on systemic pharmacotherapy, with carbamazepine and oxcarbazepine representing established first-line options (13). Both agents act through voltage-gated sodium channel blockade and achieve initial pain relief in approximately 69% to 91% of patients. However, long-term treatment is limited by dose-dependent adverse effects, progressive pharmacological tolerance, and eventual treatment failure in a substantial proportion of patients. These limitations have driven growing interest in localized interventions that directly target trigger zones and peripheral pain generators (14). Options in this category include peripheral nerve blockade, botulinum toxin type A injections, and minimally invasive ablative techniques. A more complete understanding of the mechanisms underlying trigger zone formation, combined with improved assessment tools, may ultimately support the development of precision-based and individualized treatment strategies. This review summarizes current evidence on the clinical features, pathophysiological mechanisms, and therapeutic implications of trigger zones in TN, following a narrative review methodology. Particular attention is given to the complementary roles of systemic and localized interventional approaches. Notably, distinct from general reviews that broadly recapitulate the universal pathophysiology of TN, this work treats the trigger zone not as a peripheral clinical curiosity but as a measurable, bedside-accessible intermediate phenotype bridging channel-level peripheral dysfunction and brainstem-level central sensitization. The clinical manifestations and multi-level mechanistic evidence are accordingly presented as sequential sections anchored to this shared phenotype, so that each mechanistic level is interpreted in direct reference to its corresponding trigger zone feature rather than as an independent body of TN pathophysiology. This phenotype-centered structure further allows trigger zone distribution itself to serve as a localization tool guiding branch-specific pharmacological and interventional decision-making, a translational link not directly offered by reviews that recapitulate TN mechanisms in parallel without a unifying clinical anchor. We further propose a peripheral-central synergistic sensitization model for trigger zone formation, and highlight the practical value of this framework for bedside anatomical localization and interventional strategy design.

## 2 Clinical manifestations of trigger zones

### 2.1 Distribution patterns and spatial characteristics

Trigger zones in TN are consistently distributed within the sensory territory of the affected trigeminal branch, and their anatomical location corresponds closely to the division responsible for generating pain (15). The maxillary and mandibular divisions are most frequently involved, reflecting the broader pattern of branch predominance observed in TN overall. Trigger zones associated with the maxillary division are most commonly found in the perinasal and perioral regions, including the nasal wing, upper lip, and maxillary gingival mucosa (16). Those linked to the mandibular division tend to cluster over the lower lip, chin, mandible, and corresponding intraoral mucosal surfaces. Trigger zones in the ophthalmic division territory are comparatively rare, occurring in isolation in fewer than 5% of patients, and are typically located in the supraorbital or eyebrow region when present. Trigger zones are almost exclusively unilateral in classical and idiopathic trigeminal neuralgia, and bilateral involvement is extremely uncommon. The detection of bilateral trigger zones in clinical practice should raise strong suspicion of an underlying secondary etiology. Bilateral trigger zones are far more prevalent in multiple sclerosis (MS)-associated trigeminal neuralgia than in classical or idiopathic subtypes, a clinical feature most reasonably attributed to bilateral or multifocal pontine demyelinating lesions, rather than a solitary site of unilateral neurovascular compression (17).

**Figure 1:**
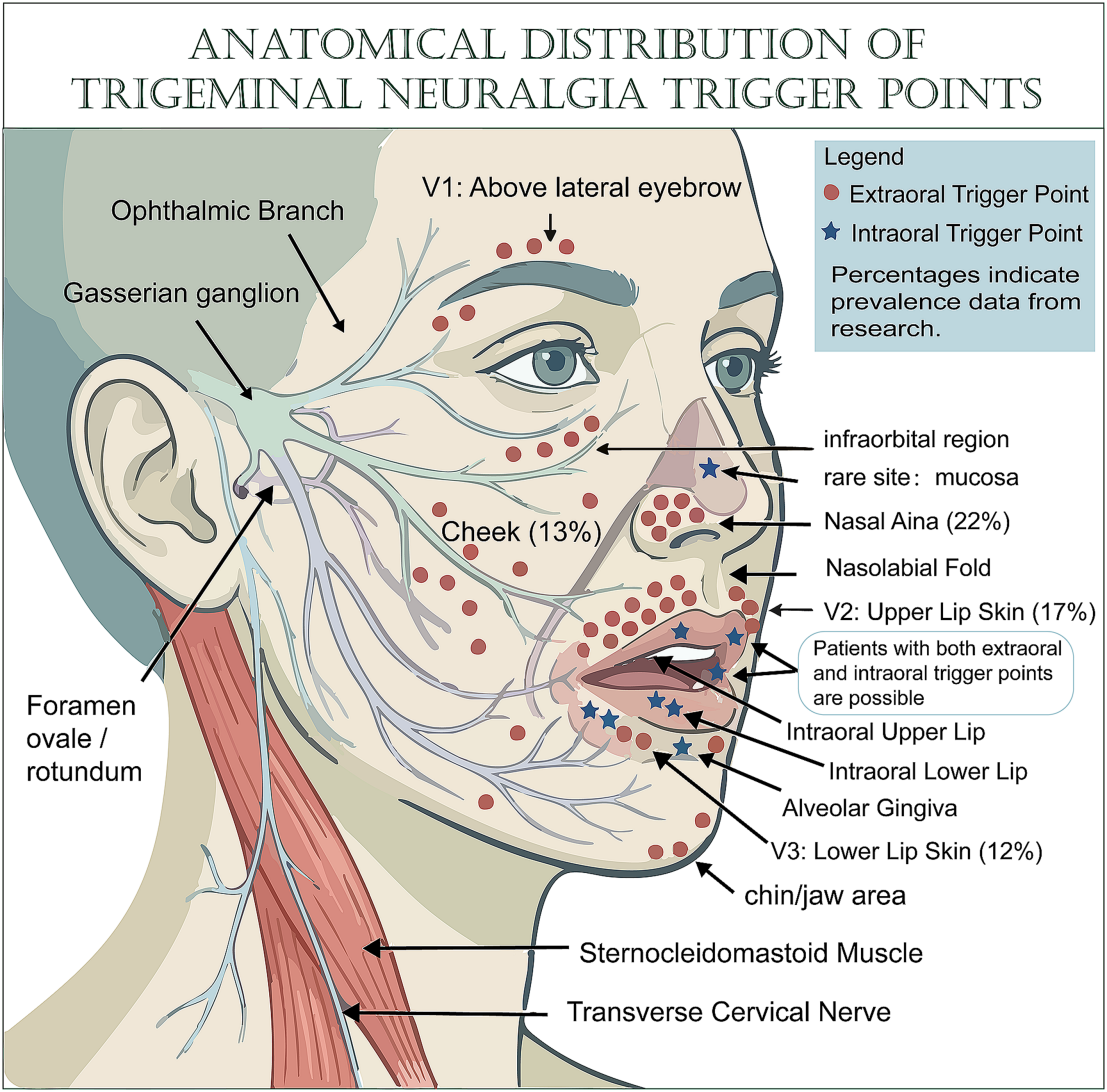
Anatomical distribution of trigger zones across the three trigeminal divisions. Red circles indicate extraoral sites and blue stars indicate intraoral sites. Percentage values are derived from the three-dimensional mapping study by Di Stefano et al. (18).

The most comprehensive mapping data available come from a dedicated investigation of 140 patients with TN in which three-dimensional facial models were used to document trigger zone locations across the full trigeminal territory (18, 19). Despite broad distribution within the trigeminal territory, trigger zones clustered preferentially in the perioral and nasal regions. The nasal wing was the single most frequent site, identified in 22% of patients (Figure 1), followed by the upper lip at 17%, the cheek at 13%, the lower lip at 12%, and the chin and alveolar gingiva each at 11%. Extraoral trigger zones were present in 79% of patients and intraoral zones in 84%, indicating that the two categories frequently coexist. This distribution pattern is thought to reflect the somatotopic organization of trigeminal sensory fibers at the root entry zone (20). At this location, maxillary and mandibular fibers occupy positions particularly vulnerable to neurovascular compression.

Regarding spatial extent, trigger zones were historically described as point-like areas with a surface area typically below 1 to 2 mm^2^, but three-dimensional sensory mapping has revealed that dimensions vary considerably across individuals (21). The term “trigger zone” is therefore more accurate than “trigger point,” as it acknowledges this spatial variability while preserving the concept of a bounded and identifiable hypersensitive region (22). The spatial characteristics of trigger zones are not static. As the disease progresses, a trigger zone may expand in area, and additional zones may emerge in neighboring or distant parts of the trigeminal territory. These changes suggest that trigger zones represent dynamic regions of neural sensitization rather than fixed anatomical structures. A single cross-sectional assessment may therefore not capture the full extent of a patient’s hypersensitive regions.

### 2.2 Relationship between trigger zones and pain attacks

The most clinically striking feature of trigger zones is their markedly low activation threshold (23). Gentle touching of the face was the most frequently reported trigger maneuver, cited by 79% of patients in the Di Stefano et al. (18) cohort. This was followed by talking in 54%, chewing in 44%, and tooth brushing in 31%. A mechanical component is present in virtually all reported triggering activities, whether involving direct cutaneous contact, airflow, or muscle movement during speech and mastication. In contrast, slow sustained pressure and thermal stimuli are substantially less effective at provoking attacks.

An important diagnostic feature of trigger zone-evoked pain is that the site of stimulation and the site of perceived pain do not always coincide (24). In most patients, pain is experienced within the same trigeminal division as the trigger zone and radiates in a pattern consistent with the affected branch. However, in a subset of patients, pain may be referred beyond the immediate vicinity of the trigger zone or across divisional boundaries. This spatial dissociation between the stimulated area and the perceived painful region distinguishes trigger zone-evoked pain in TN from conventional mechanical allodynia. It has been interpreted as evidence that central processing mechanisms contribute to the final pain percept.

Pain provoked by trigger zone stimulation also exhibits abnormal spatiotemporal summation. Sustained low-intensity stimulation may eventually reach a threshold sufficient to precipitate an attack, and increasing the frequency or intensity of repeated stimuli can reduce the latency to pain induction. This pattern of abnormal temporal summation, in which repeated activation of low-threshold mechanoreceptive Aβ fibers builds toward an attack, differs fundamentally from normal nociceptive summation mediated by Aδ and C fibers (25). It provides clinical evidence for central sensitization within the trigeminal system. Following an attack, a refractory period during which the same stimulus fails to reproduce pain is consistently observed, lasting from several seconds to several minutes. This refractory period is a clinically distinctive feature of TN not observed in other forms of mechanical allodynia, and its presence is a useful diagnostic marker in the clinical examination.

### 2.3 Clinical and objective assessment of trigger zones

The bedside identification of trigger zones relies on gentle stimulation of successive facial areas using a cotton swab or the examiner’s fingertip (26). The aim is to reproduce a typical paroxysmal attack. When stimulation of a specific area reliably precipitates pain consistent with the patient’s habitual attacks, that region is identified as a trigger zone. The examination is straightforward and requires no specialized equipment. A clinically important observation is that TN patients typically guard trigger regions carefully and keep the affected side of the face still (27). Male patients may present with an unshaved patch of skin overlying the trigger zone, providing an inadvertent visual guide to the hypersensitive area before formal examination begins.

Despite the simplicity of bedside examination, studies applying quantitative sensory testing have consistently revealed subclinical sensory changes that routine neurological assessment misses. Flor and colleagues applied the standardized protocol of the German Research Network on Neuropathic Pain to 48 patients with idiopathic TN and 27 matched controls. They found deficits in warm and cold detection thresholds in both affected and unaffected nerve branches (28). A blinded quantitative sensory testing study by Younis and colleagues further demonstrated that TN patients without any detectable deficit on routine neurological examination showed generalized subclinical hypoesthesia more pronounced on the symptomatic side (29). Bilateral thermal and mechanical hyperalgesia was also identified in both the face and the hand. These findings indicate that sensory disturbance in TN extends well beyond the immediate territory of the trigger zone and reflects a broader alteration in trigeminal sensory processing.

High-resolution ultrasound operating at frequencies of 10 to 18 MHz allows visualization of superficial trigeminal nerve branches and adjacent soft tissue structures in real time (30). In patients with refractory TN, ultrasound examination of trigger zone regions has in some cases revealed focal muscle fasciculations and subtle soft tissue hyperactivity not detectable by palpation (31). These findings provide a degree of objective anatomical grounding to trigger zone locations that have historically been defined purely on the basis of patient report. The integration of quantitative sensory testing and high-resolution ultrasound into trigger zone assessment represents a promising direction for moving beyond subjective characterization toward reproducible, objective measurement. Standardized protocols for clinical and research use, however, remain to be established. These trigger zone-evoked attack characteristics are best documented in classical and idiopathic TN. In MS-associated TN, paroxysmal attacks more frequently coexist with a persistent background ache and involve more than one trigeminal division simultaneously, a pattern less typical of classical TN (7).

## 3 Pathophysiological mechanisms underlying trigger zone formation

Trigger zones represent one of the most diagnostically distinctive features of TN, and are formally recognized as a core diagnostic criterion in the ICHD-3 classification. It is important to clarify that trigger zones are not an independent pathological entity separate from underlying trigeminal nerve dysfunction, but rather a focal clinical phenotype shaped by the combined effects of root-level pathology, local peripheral terminal modifications, and central processing alterations. Nonetheless, they warrant dedicated mechanistic interpretation rather than being dismissed as a trivial epiphenomenon, given their core diagnostic value, distinct spatial specificity, active role in sustaining pathological circuits, and utility as independent therapeutic targets. While the exact mechanisms remain a subject of ongoing investigation, current studies propose to elucidate the maintenance and dynamic evolution of trigger zone hypersensitivity at three hierarchical levels: peripheral excitability regulation, local mechanical modulation, and central signal amplification. It should be noted that many of these models, particularly those involving peripheral-central synergistic interactions, are currently based on a combination of preclinical evidence and clinical inference, and thus require further validation to be considered definitive drivers of trigger zone formation. These three processes are not mutually exclusive but interact across multiple levels of the nervous system to establish and maintain the hypersensitive regions that define trigger zones in clinical practice.

### 3.1 Peripheral nerve hyperexcitability

#### 3.1.1 Role of voltage-gated sodium channels

Voltage-gated sodium channels are the molecular determinants of neuronal membrane excitability and the primary generators of action potentials in sensory afferents (32). Following nerve injury or sustained mechanical stress, their expression and functional state are disrupted in trigeminal sensory neurons (33). It is inferred that this disruption shifts these cells toward a persistently hyperexcitable state and lowers the threshold at which peripheral stimuli generate propagating electrical activity.

Accordingly, findings related to Nav1.9 specifically should be interpreted with a clear distinction between experimental observations and mechanistic inferences. Among sodium channel subtypes implicated in TN, Nav1.9 has attracted the most research attention in the pathophysiology of trigger zones. Compared with other well-characterized subtypes, the biophysical properties and anatomical distribution of Nav1.9 show the highest degree of alignment with the core phenotypic features of trigger zones, making it the most prominent candidate molecule in investigations of peripheral mechanisms underlying trigger zones.

**Figure 2:**
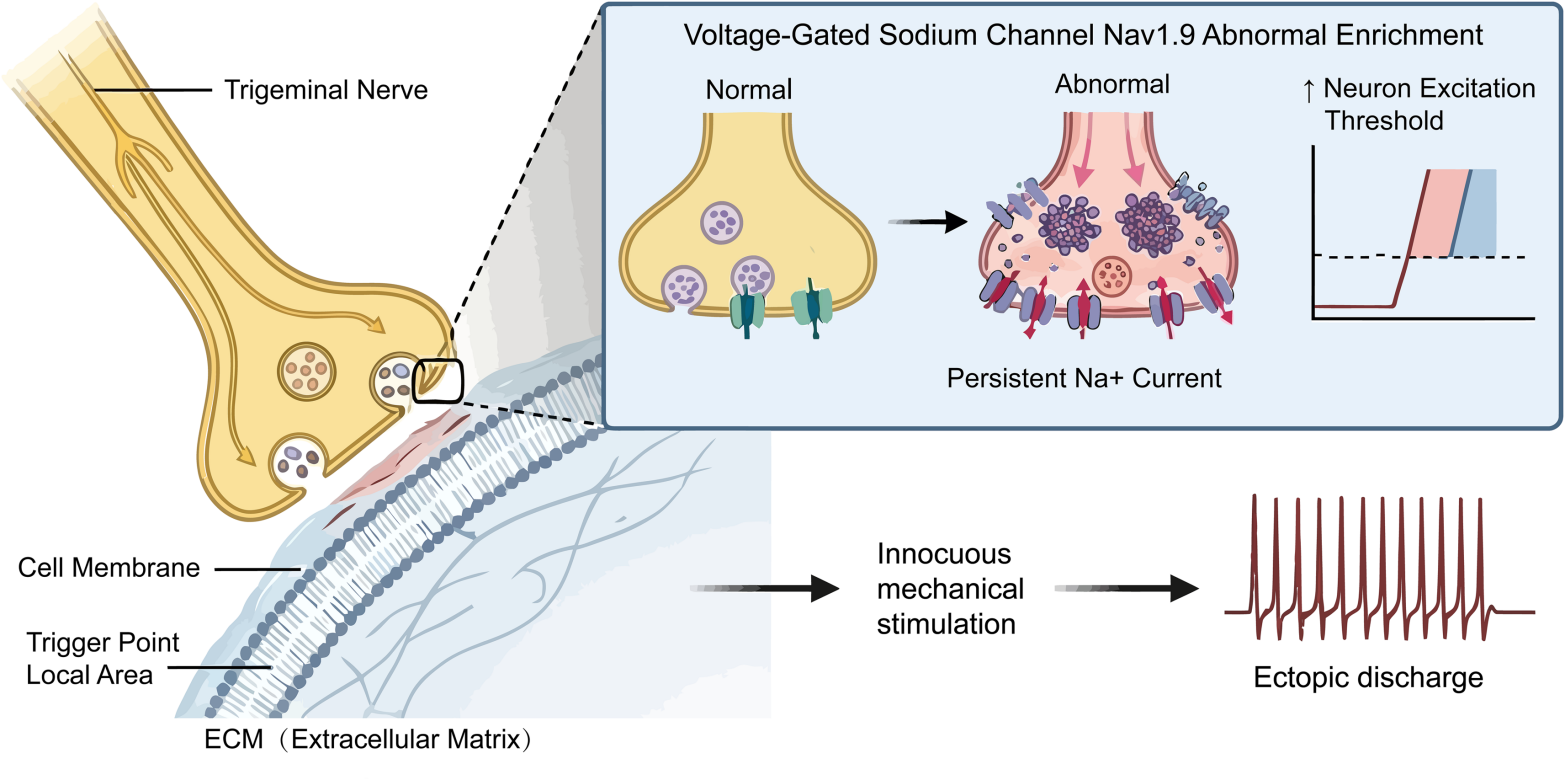
Proposed model of Nav1.9-driven peripheral hyperexcitability at the trigeminal sensory terminal. Abnormal channel accumulation is hypothesized to generate a persistent inward sodium current, lowering the firing threshold such that innocuous mechanical stimulation produces repetitive ectopic discharge.

Functionally this tetrodotoxin-resistant channel generates a persistent, non-inactivating inward sodium current at subthreshold membrane potentials (Figure 2), a well-documented biophysical property that sustains partial neuronal depolarization. In the context of Nav1.9 upregulation, this property may allow even mild mechanical stimuli to reach the threshold for burst firing. Nav1.9 is preferentially expressed in the sensory territories of the maxillary and mandibular trigeminal divisions. This distribution corresponds closely with the anatomical regions where trigger zones most commonly develop (34). Preclinical animal model studies using infraorbital nerve constriction have further demonstrated that Nav1.9 activity is required for the development of mechanical hypersensitivity in the orofacial territory. Extrapolating these findings to human trigeminal neuralgia, Nav1.9 dysregulation has been proposed as a key contributor to the reduced activation threshold of trigger zones, though this causal association has not been directly verified in human TN tissue, and larger confirmatory human tissue studies are still needed.

Nav1.7, expressed at high density in trigeminal ganglion neurons, functions as a threshold channel that amplifies small depolarizing stimuli otherwise insufficient to generate a spike. Gain-of-function mutations in its encoding gene produce inherited paroxysmal pain syndromes that parallel the episodic nature of TN pain (35). These monogenic channelopathies feature abrupt, shock-like pain episodes clinically reminiscent of classic trigeminal neuralgia. Downregulation of Nav1.7 observed in gingival tissue from TN patients has been interpreted as a chronic adaptive reorganization of channel expression in the setting of repeated paroxysmal activity. Nav1.8, with its rapid repriming kinetics, supports high-frequency repetitive firing and undergoes redistribution from injured to neighboring uninjured axons following nerve damage, conferring hyperexcitability on fiber populations that were previously functioning normally (36). Nav1.3, an embryonic channel re-expressed after nerve injury, has been confirmed as upregulated in gingival tissue from TN patients and contributes to ectopic burst discharge through its rapid repriming and low activation threshold properties (37). Taken together, the altered expression of these four channel subtypes produces a significant phenotypic shift in trigeminal sensory neurons, moving the membrane from a state of normal regulation to one of persistent subthreshold depolarization and readiness for synchronized burst firing. Importantly, the strength of supporting evidence varies substantially across subtypes. The therapeutic efficacy of systemic sodium channel blockers in TN represents a clinically established conclusion. By contrast, the specific contribution of individual subtypes to trigger zone formation remains at the level of mechanistic inference, with Nav1.9 in particular lacking direct confirmatory evidence from human TN tissue.

#### 3.1.2 Demyelination, axonal damage, and ectopic discharge

The pathological substrate linking neurovascular compression to trigger zone formation is focal demyelination at the trigeminal root entry zone, specifically at the Obersteiner-Redlich zone where oligodendrocyte-derived central myelin transitions to Schwann cell-derived peripheral myelin (38). Central myelin is structurally less resistant to sustained mechanical deformation, making this transitional segment selectively vulnerable to pulsatile vascular indentation. Histopathological examination of trigeminal nerve roots obtained during microvascular decompression has consistently demonstrated focal demyelination at the compression site. Demyelinated axons lie in close apposition with an absence of intervening glial processes (39).

Demyelinated axons develop spontaneous ectopic discharge and abnormal mechanosensitivity, generating action potentials in response to mechanical deformation that would be entirely innocuous in a healthy nerve (40). The most clinically significant consequence of close membrane apposition between demyelinated fibers of different functional classes is ephaptic transmission. In this process, electrical current generated in a tactile Aβ fiber spreads laterally to an adjacent nociceptive Aδ fiber without synaptic contact. This mechanism provides a direct anatomical and electrophysiological explanation for the defining feature of TN trigger zones: light mechanical stimulation of a restricted facial area precipitating severe paroxysmal pain.

The ignition hypothesis proposed by Devor, Amir, and Rappaport offers a theoretical framework that integrates these histopathological and electrophysiological observations (41). Within this proposed model, a subpopulation of injured trigeminal afferents is thought to develop a hair-trigger threshold for burst discharge while remaining electrically silent under baseline conditions. Tactile input arriving along Aβ fibers from a trigger zone is proposed to ignite this primed population through ephaptic cross-excitation. The resulting rapidly amplifying synchronized discharge is posited to underlie the explosive, shock-like quality of the pain attack (42). The post-attack refractory period reflects the time required for ionic gradients to be restored before the neuronal population can re-enter its hyperexcitable state. The somatotopic correspondence between root entry zone demyelination and peripheral trigger zone location reflects the preferential vulnerability of maxillary and mandibular fibers to superior cerebellar artery compression. This vulnerability may partly explain the predominance of perioral and nasal trigger zones in the clinical population.

### 3.2 Muscle-nerve interactions

#### 3.2.1 Mechanical sensitization via facial and masticatory muscle activity

**Figure 3:**
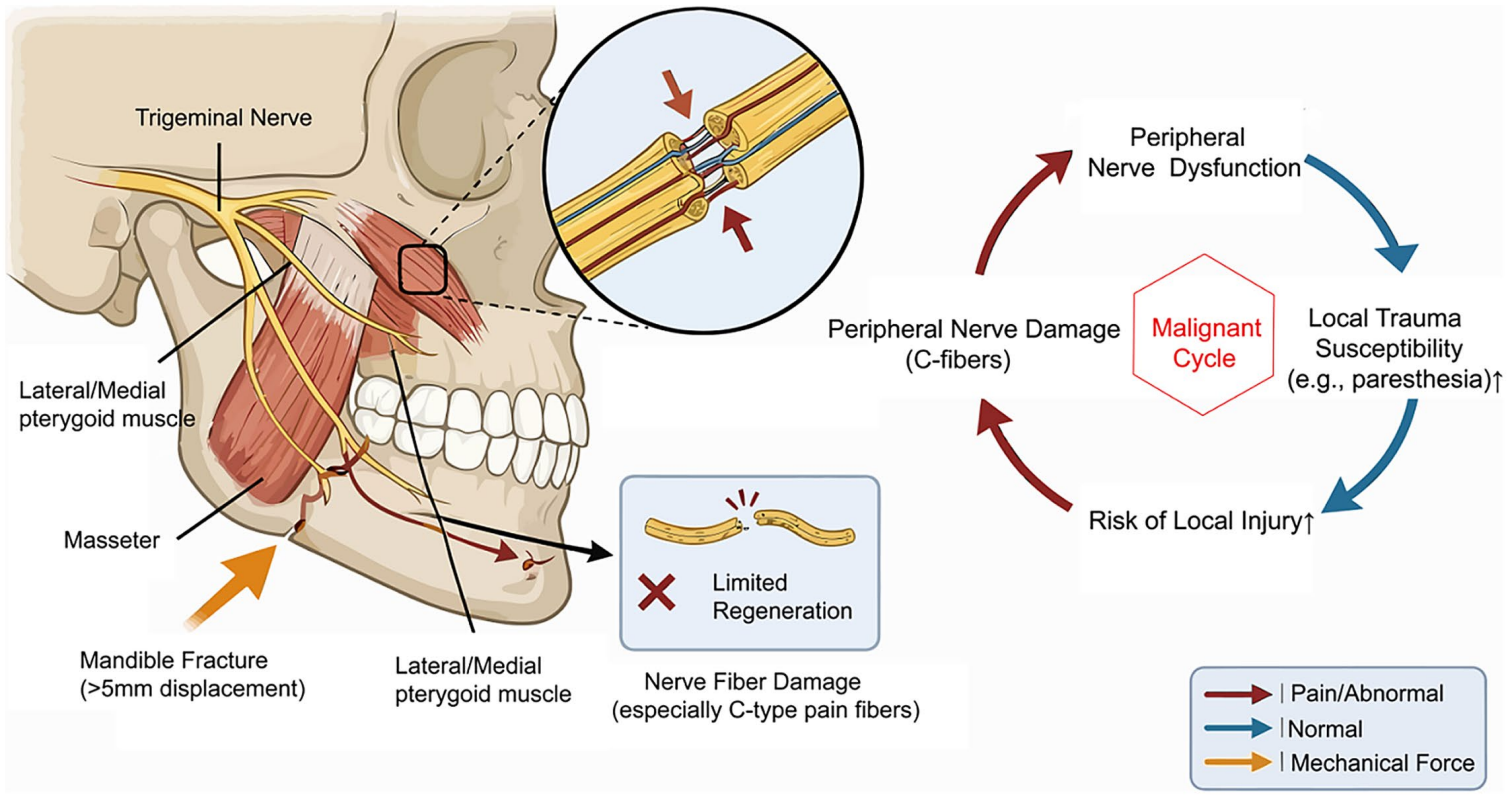
Proposed vicious cycle linking C-fiber damage, impaired regeneration, and escalating local injury susceptibility under masticatory muscle mechanical stress. Dark red arrows indicate pain and abnormal signaling pathways, blue arrows indicate normal signaling, and orange arrows indicate mechanical force vectors.

While the exact etiology is yet to be fully elucidated, a widely accepted hypothesis posits that local mechanical stimulation at the trigger zone acts as a threshold-lowering stimulus, triggering ectopic discharges in already hyperexcitable afferent fibers. Beyond intrinsic neural abnormalities, the mechanical environment surrounding peripheral trigeminal nerve endings in the facial soft tissues has been proposed as a contributing factor to trigger zone formation, although the supporting evidence remains largely indirect and awaits further prospective validation (43). Facial expression muscles and masticatory muscles undergo repeated contractions throughout each waking hour. Trigeminal sensory branches coursing through or adjacent to these muscles are therefore exposed to compressive and tensile forces with every movement cycle. In the context of an already sensitized trigeminal nerve, physiologically normal muscle activity may provide sufficient mechanical stimulus to lower the activation threshold of nearby nerve endings or precipitate ectopic discharge (Figure 3).

**Figure 4:**
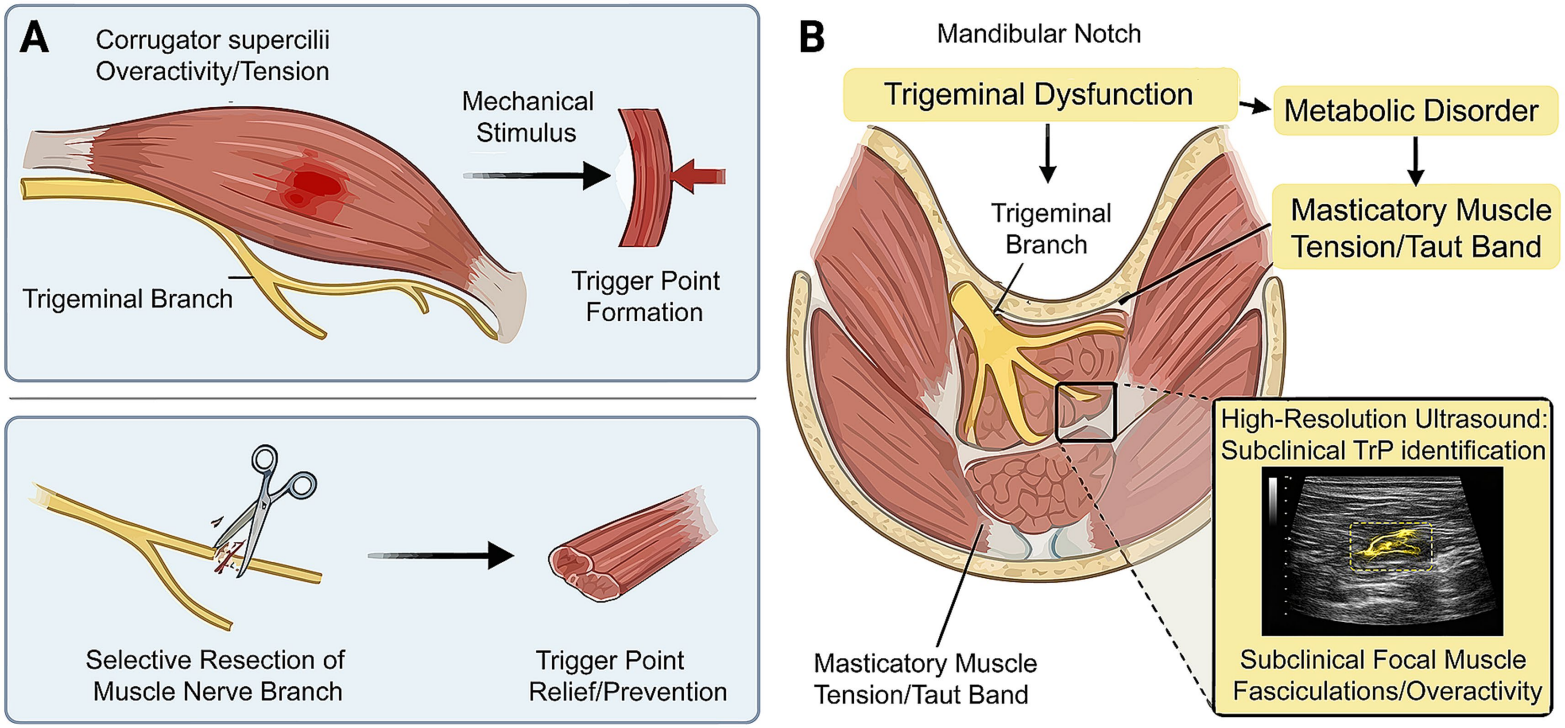
Two proposed muscle-nerve interaction pathways contributing to trigger zone formation. **(A)** Shows that *corrugator supercilii* overactivity imposes mechanical stress on the adjacent trigeminal branch, and selective nerve branch resection achieves sustained relief. **(B)** Shows that masticatory muscle tension produces subclinical fasciculations detectable by high-resolution ultrasound.

The best-documented example of this pathway involves the corrugator supercilii muscle and the supraorbital and supratrochlear branches of the ophthalmic division. These nerve branches pass through or immediately adjacent to the corrugator supercilii as they ascend toward the forehead, and repeated contraction of this muscle may impose chronic low-grade mechanical compression on sensitized sensory fibers. In a single-center case series of 15 patients with purely paroxysmal TN confined to the supraorbital and supratrochlear territories, Gualdi and colleagues reported that selective surgical denervation of the corrugator supercilii yielded significant and sustained relief of trigger zone-related pain (Figure 4). All patients in this series had previously responded to botulinum toxin type A injections targeting the same muscle (44). These therapeutic outcomes provide indirect but meaningful evidence that local muscle activity can modulate the excitability of adjacent sensory nerve endings.

A small observational study using high-resolution ultrasound examination of trigger zone regions in patients with refractory TN has revealed focal muscle fasciculations and subtle soft tissue hyperactivity not detectable by palpation (45). The spatial correspondence between these ultrasound findings and patient-reported trigger zone locations supports the plausibility of a mechanistic link. Notably, the evidence base for this pathway remains limited and predominantly indirect. To date, supporting data are largely derived from small single-arm clinical series, observational imaging findings and indirect inferences from interventional outcomes, with no large-scale prospective studies confirming a causal relationship. The muscle-nerve interaction pathway should therefore be interpreted as a supplementary, hypothesis-driven mechanism rather than an established core driver of trigger zone formation. Whether muscle hyperactivity drives neural sensitization, or whether sensitized nerves sustain local muscle activity through aberrant efferent-like signaling, remains to be established prospectively.

#### 3.2.2 Conceptual differentiation from myofascial trigger points

**Figure 5:**
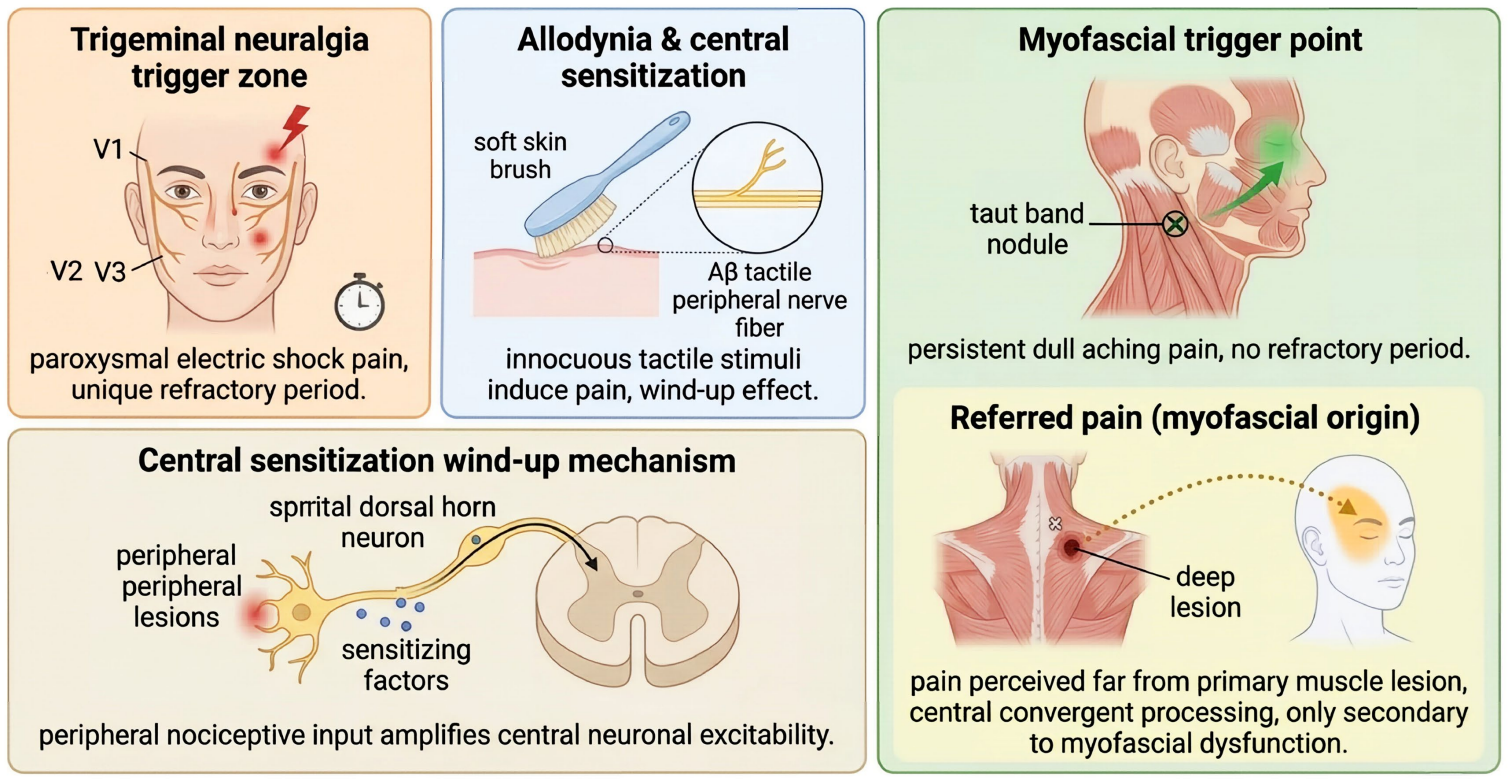
Comparative schematic of core facial pain phenotypes differentiating trigeminal neuralgia trigger zone, mechanical allodynia, myofascial trigger point and its secondary referred pain. Distinct diagnostic traits including pain quality, refractory period, stimulus-pain spatial correspondence and pathological substrate are visually separated to facilitate discrimination, consistent with the textual comparison summarized in Table 1.

The terminological overlap between TN trigger zones and myofascial trigger points is a persistent source of diagnostic ambiguity in craniofacial pain practice. The two entities differ fundamentally in pathological substrate, pain quality, temporal behavior, and treatment response, as summarized in Table 1. Myofascial trigger points originate from primary pathological changes within the muscle tissue itself, with nociceptive signals arising secondarily from local metabolic disturbance and sensitization of intramuscular nerve endings. TN trigger zones, by contrast, represent abnormal excitability of trigeminal sensory afferents whose cutaneous or mucosal innervation territory constitutes the hypersensitive region; the overlying skin or mucosa is not intrinsically diseased. Clinically, TN trigger zones respond to minimal light touch with an immediate explosive attack followed by a refractory period. Myofascial pain evoked by palpation is dull and aching, and is not followed by a refractory interval. The muscle contribution discussed above is a modifying influence on a disorder whose pathological foundation lies in the trigeminal sensory system. This distinction must be preserved in both clinical reasoning and research design (Figure 5).

**Table 1:** Key distinctions between TN trigger zones and myofascial trigger points.

Feature	TN trigger zones	Myofascial trigger points
Pathological substrate	Peripheral trigeminal sensory afferent hyperexcitability; demyelination and ectopic discharge	Taut band within skeletal muscle; dysfunctional neuromuscular junction
Evoked pain quality	Explosive, electric shock-like; abrupt onset and termination	Dull, aching; gradual onset; persists after stimulation
Refractory period	Present	Absent
Response to nerve block	Abolishes trigger response and pain radiation	Does not reliably eliminate referred pain
Primary treatment target	Sensitized trigeminal afferents	Muscle pathology

### 3.3 Peripheral-central synergistic sensitization

#### 3.3.1 Central sensitization within the trigeminal brainstem complex

Evidence suggests that central sensitization within the trigeminal brainstem complex potentially contributes to the amplification of innocuous tactile inputs into pain, a process that warrants further investigation to confirm its role in human TN pathophysiology. Persistent abnormal afferent input generated by ectopic discharge and ephaptic cross-excitation is believed to create a sustained barrage of nociceptive signals arriving at the trigeminal brainstem complex (46). These signals are thought to induce functional and structural changes that lower the threshold for pain generation and broaden the sensory territory within which stimulation can provoke an attack. This central sensitization actively amplifies nociceptive signaling and contributes to the progressive nature of TN as the disease evolves.

Within the trigeminal brainstem complex, wide dynamic range neurons receive convergent input from both low-threshold mechanoreceptive Aβ fibers and high-threshold nociceptive Aδ and C fibers (47). Under normal conditions, these neurons respond only modestly to innocuous mechanical stimuli. Following sustained peripheral ectopic discharge, their activation threshold falls, their receptive fields expand, and they generate exaggerated responses to inputs that previously produced only subthreshold activity. As well documented in preclinical pain models, repeated high-frequency afferent input promotes NMDA receptor activation at second-order neurons, initiating calcium influx and intracellular signaling cascades linked to long-term potentiation of synaptic efficacy (48). This pathway is widely inferred to operate analogously in the human trigeminal brainstem in TN, though direct confirmatory evidence in patient tissue remains limited. Substance P and calcitonin gene-related peptide, released during repeated paroxysmal attacks, further enhance excitatory transmission at the brainstem level.

**Figure 6:**
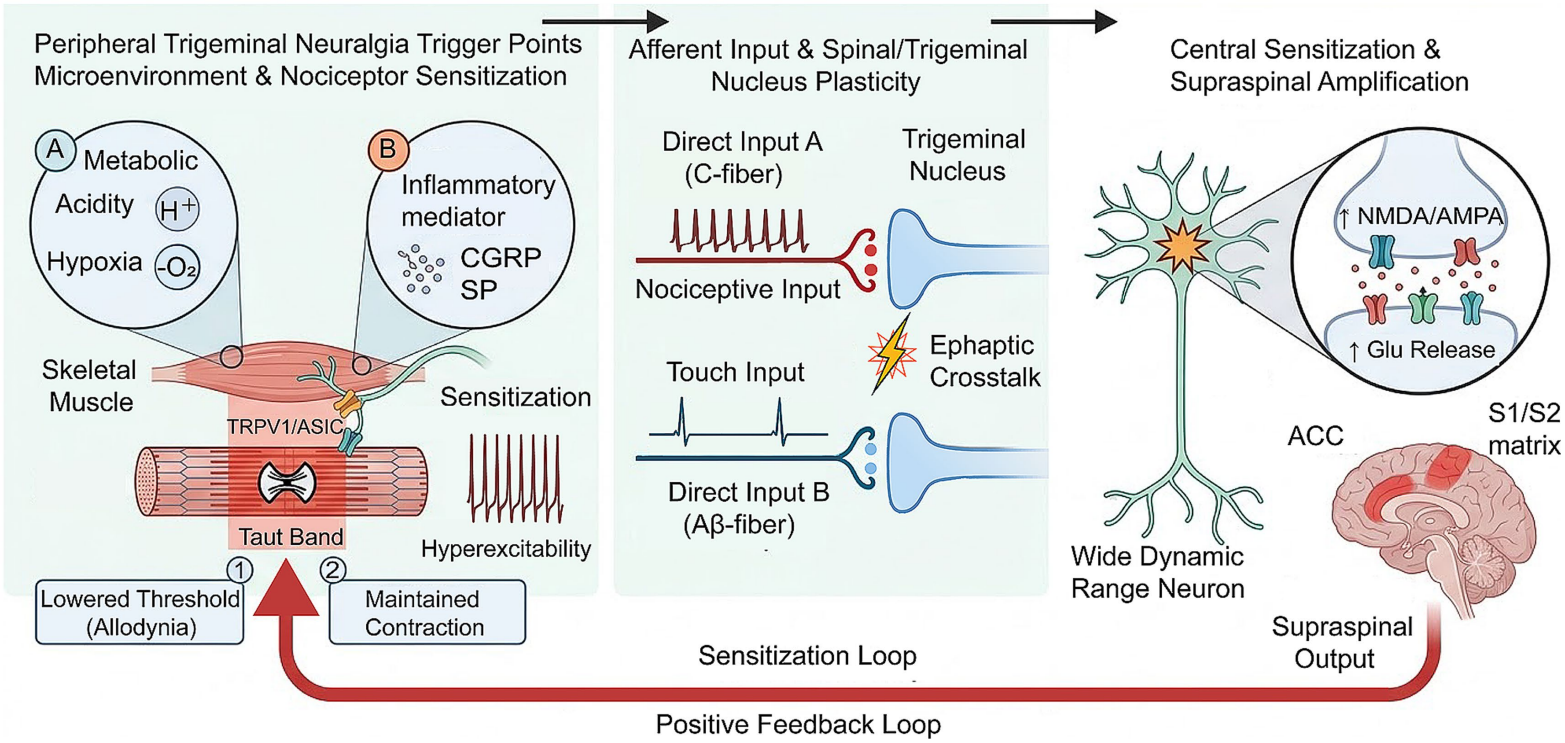
Conceptual integrated model of peripheral-to-central sensitization and the positive feedback loop sustaining trigeminal neuralgia. Peripheral nociceptor sensitization and ephaptic crosstalk at the trigeminal nucleus drive wide dynamic range neuron activation, supraspinal amplification, and a self-reinforcing cycle that perpetuates trigger zone hypersensitivity.

The electrophysiological consequence most relevant to trigger zone formation is the aberrant processing of Aβ fiber input that emerges from this sensitized state (49). Electrophysiological studies in TN patients have confirmed that trigger zone-evoked pain requires repeated co-activation of low-threshold Aβ fibers and high-threshold Aδ fibers. This pattern is fundamentally different from normal temporal summation mediated by Aδ and C fibers alone. This abnormal co-activation depends specifically on the altered central processing state established by ongoing peripheral input. Additionally, quantitative sensory testing has revealed that sensory abnormalities in TN patients extend to unaffected branches on the ipsilateral side and in some cases to the contralateral face and hand (50). This pattern indicates a global increase in central nervous system excitability that exceeds what could be explained by focal peripheral injury alone (Figure 6).

#### 3.3.2 Functional neuroimaging evidence for central involvement

Functional neuroimaging studies have directly captured the abnormal central processing of trigger zone stimulation in living patients, providing objective confirmation of the central sensitization mechanisms described above. The most informative task-based fMRI study was conducted by Moisset and colleagues in 15 patients with classical TN, a small, single-center, cross-sectional cohort. The study applied identical light tactile stimuli to both the affected trigger zone and the homologous contralateral region (51). Non-painful stimulation of the trigger zone already produced abnormal activation extending beyond the primary and secondary somatosensory cortices to include the thalamus, insula, premotor cortex, prefrontal areas, putamen, and hippocampus. When the same stimulus precipitated a pain attack, activation extended further to the spinal trigeminal nucleus, deeper brainstem structures, and the anterior cingulate cortex (52). After successful surgical decompression, stimulation of the previously affected side produced activation confined to S1 and S2, closely resembling the healthy pattern. This pre-post pattern is consistent with peripheral input driving the abnormal supraspinal activation, but because the cohort was small and uncontrolled for factors such as time since surgery and concurrent medication change, reverse causality and confounding cannot be excluded, and the finding awaits independent replication in a larger, controlled sample.

The involvement of the spinal trigeminal nucleus specifically during painful stimulation confirms at the imaging level the abnormal wide dynamic range neuron processing proposed on electrophysiological grounds. Structural neuroimaging has further shown that the central reorganization associated with TN extends to gray matter morphology. Voxel-based morphometry studies have identified volume reductions in the anterior cingulate cortex, parahippocampus, and temporal lobe that correlate positively with disease duration (53). A meta-analysis of structural and functional neuroimaging studies in TN identified consistent alterations in the thalamus as the most reproducible finding across studies (54). This suggests that the thalamus occupies a particularly vulnerable position in the central consequences of repeated trigeminal paroxysms. Taken together, peripheral neural dysfunction within trigger zones is proposed to act as an initiating factor that drives central sensitization. Central sensitization in turn is hypothesized to amplify nociceptive signaling, lower the threshold for stimulus-induced pain, and contributes to the persistence and progressive nature of trigger zone hypersensitivity, though current cross-sectional imaging evidence alone cannot establish this directionality.

### 3.4 Limitations of current evidence and alternative interpretations

The current understanding of peripheral hyperexcitability and central sensitization in TN is predominantly built on preclinical animal models, *in vitro* electrophysiological data, and small-scale human investigations. Most human mechanistic studies include fewer than 20 participants, adopt single-center cross-sectional designs, and lack longitudinal follow-up. Across the field, key mechanistic studies generally lack pre-registered protocols, blinded outcome assessment, and independent external replication, and no standardized formal risk-of-bias framework has been established for this body of literature. Within the neuroimaging subset of this literature specifically, an independent systematic review likewise characterizes the available studies as predominantly small, cross-sectional, and single-center (55). The relative length of discussion devoted to each line of evidence does not correspond to its methodological rigor or evidentiary strength.

Beyond these general methodological constraints, several specific findings further complicate the proposed model and warrant explicit acknowledgment rather than omission. First, a subset of patients with classical TN and clearly demonstrated neurovascular compression continue to experience trigger zone-evoked pain after technically successful microvascular decompression, a pattern difficult to reconcile with a purely peripherally-driven model and suggestive of a persistent, at least partially autonomous, central component in some individuals (56, 57). Second, idiopathic TN occurring without any identifiable neurovascular compression or visible demyelinating lesion has been reported in a meaningful minority of patients, raising the possibility that mechanisms other than root-entry-zone demyelination and ephaptic transmission can independently generate the trigger zone phenotype in this subgroup (58). Third, expression and electrophysiological findings for individual sodium channel subtypes, particularly Nav1.9, have not been uniformly replicated across studies, and some investigations have failed to detect the expected upregulation in human tissue, suggesting that the sodium channelopathy model may apply more consistently to some patients than to others rather than representing a universal mechanism (59). Fourth, although the corrugator supercilii denervation findings (44) and the cross-sectional fMRI normalization pattern (51) are frequently cited as supporting evidence for the muscle-nerve interaction and peripherally-driven central sensitization, respectively, neither has been independently replicated in larger or different cohorts.

Taken together, these observations indicate that the peripheral-central synergistic model, while supported by a convergent body of evidence, is unlikely to apply uniformly across all TN phenotypes, and that an independent or partially autonomous central contribution cannot be excluded in at least some patients. In light of both the methodological constraints and the conflicting findings discussed above, the proposed peripheral-central synergistic sensitization model should be interpreted as an evidence-informed theoretical framework for integrating available findings, rather than a conclusively validated causal pathway. To clarify the evidentiary basis of all mechanisms covered in this section, we systematically summarize these pathophysiological mechanisms and their corresponding evidence grades in Table 2.

**Table 2:** Evidence strength summary for proposed trigger zone pathophysiological mechanisms.

Proposed mechanism	Evidence category	Key supporting evidence	Key uncertainty / limitation
Neurovascular compression-induced demyelination at the root entry zone (Obersteiner-Redlich zone)	Established clinical evidence	Consistently demonstrated on histopathology of nerve roots obtained during microvascular decompression; well-replicated across surgical series	Does not explain trigger zones in idiopathic TN without demonstrable compression or in patients with persistent pain after successful decompression
Sodium channel dysfunction as a peripheral driver (supported by therapeutic efficacy of channel blockers)	Established clinical evidence	Multiple placebo-controlled RCTs and large real-world cohorts; first-line status across guidelines	Confirms global channel involvement but does not isolate the specific channel subtype(s) responsible for trigger zone hyperexcitability
Central sensitization of wide dynamic range neurons in the trigeminal brainstem complex (abnormal Aβ/Aδ co-activation, temporal summation)	Moderate evidence	Electrophysiological studies in TN patients confirm abnormal Aβ-Aδ co-activation and temporal summation distinct from normal nociceptive processing	Mechanistic link to NMDA-mediated long-term potentiation is extrapolated from preclinical pain models rather than directly confirmed in human brainstem tissue
Functional and structural neuroimaging changes accompanying trigger zone stimulation (cortical/subcortical activation, thalamic and gray-matter alterations)	Moderate evidence	Task-based fMRI showing stimulation- and surgery-dependent activation changes; voxel-based morphometry and meta-analytic thalamic involvement	Based predominantly on small, single-center, cross-sectional cohorts (e.g., n = 15); pre-post fMRI pattern not independently replicated and cannot establish causal directionality
Ephaptic cross-excitation between demyelinated Aβ and Aδ/nociceptive fibers (the “ignition hypothesis”)	Moderate evidence	Histopathological apposition of demyelinated fibers without intervening glia is well-documented across surgical series; the ignition hypothesis provides the most coherent mechanistic interpretation of this finding and of the paroxysmal attack phenotype	The unifying ignition model itself remains a theoretical framework integrating histopathological and electrophysiological observations rather than a directly demonstrated mechanism in vivo
Sodium channel subtype-specific contributions (Nav1.9, Nav1.7, Nav1.8, Nav1.3)	Moderate evidence (Nav1.3); Limited evidence (Nav1.9, Nav1.7, Nav1.8)	Nav1.3 upregulation confirmed in human gingival tissue from TN patients; Nav1.7 downregulation observed in human tissue; monogenic Nav1.7 channelopathies parallel TN phenotype	Nav1.9 contribution relies on preclinical rodent models and has not been directly verified in human TN tissue, with inconsistent replication; Nav1.8 redistribution is inferred from general nerve-injury models rather than TN-specific data
Mechanical sensitization via facial and masticatory muscle activity (muscle-nerve interaction)	Limited evidence	Case series of corrugator supercilii denervation relieving trigger zone pain; ultrasound findings of focal muscle fasciculations near trigger zones	Evidence derives from small, single-center series and observational imaging; no large-scale prospective studies; direction of causality (muscle to nerve vs. nerve to muscle) unresolved
Peripheral-central synergistic sensitization as a unifying bidirectional feedback model	Hypothesis-generating	Conceptually integrates peripheral ectopic discharge with central sensitization and is consistent with available preclinical evidence and clinical observational data	Explicitly proposed as an evidence-informed theoretical framework rather than a conclusively validated causal pathway; does not apply uniformly across all TN phenotypes

## 4 Clinical application: trigger zone-guided therapeutic decision-making

The distribution of trigger zones in a given patient carries direct diagnostic utility that shapes treatment planning before any intervention is selected. Trigger zones mapping to the nasal wing or upper lip implicate the maxillary division, while those over the chin or mandibular gingiva point to the mandibular division. This localization information guides not only pharmacological targeting but also the selection of nerve block injection sites, botulinum toxin injection territories, and neuromodulation targets. The two principal treatment categories, systemic pharmacotherapy and trigger zone-targeted local interventions, are summarized alongside their mechanisms, efficacy data, and clinical indications in Table 3.

**Table 3:** Overview of treatment strategies in TN.

Treatment	Mechanism	Key efficacy	Main risks	Indication
Carbamazepine	Voltage-gated Na^+^ channel blockade	Responder rate 69–75%	Dizziness, hyponatremia, hepatotoxicity	First-line, all subtypes
Oxcarbazepine	Voltage-gated Na^+^ channel blockade	Responder rate 88–91%; fewer side effects than carbamazepine	Hyponatremia; fewer drug interactions	First-line; preferred in polypharmacy
Lamotrigine/Baclofen	Na^+^ channel blockade/GABA-B agonism	Limited RCT evidence; Level C	Sedation; slow titration required	Add-on when first-line agents fail
Gabapentin/Pregabalin	α2δ calcium channel modulation	Comparable efficacy to carbamazepine; better tolerability	Dizziness, somnolence	Add-on; concomitant continuous pain
Lacosamide	Enhances slow inactivation of voltage-gated Na^+^ channels	Oral: responder rate ~66% in refractory TN; IV: pain relief in ~78% of acute exacerbations	Somnolence, dizziness; rare cardiac conduction effects	Add-on (oral) or rescue therapy for acute exacerbations (IV)
Peripheral nerve block	Reversible afferent conduction blockade	Immediate relief in majority; duration hours to weeks	Transient sensory disturbance; requires repeat injection	Bridging analgesia; diagnostic localization
Botulinum toxin type A	Inhibits neuropeptide release; reduces peripheral sensitization	Responder rate 65–76%; RR 2.87 vs. placebo	Transient facial asymmetry; self-resolving	Inadequate or intolerable pharmacotherapy
Radiofrequency thermocoagulation	Selective thermal disruption of nociceptive fibers	Immediate relief 90–100%; recurrence ~29–43% at 3–4 years	Facial hypoesthesia; rare dysesthesia	Refractory TN; unsuitable for MVD
Combined systemic and local	Complementary peripheral and central modulation	Greater pain reduction and lower drug dose than monotherapy	Additive adverse effect profile	Inadequate monotherapy response

### 4.1 Systemic pharmacotherapy

#### 4.1.1 First-line sodium channel blockers

Systemic pharmacotherapy remains the foundation of TN management, and voltage-gated sodium channel blockers are the established first-line agents (60). Their rationale is mechanistically grounded in the peripheral neural hyperexcitability described above. By stabilizing neuronal membrane potentials and suppressing ectopic discharges in trigeminal afferents, these drugs address the peripheral mechanism that initiates the trigger zone activation cascade.

Carbamazepine is the best-studied agent, with the longest clinical track record and regulatory approval specifically for TN (61). It exerts frequency-dependent sodium channel blockade, preferentially suppressing the rapid repetitive firing characteristic of ectopic discharge (62). Two placebo-controlled randomized controlled crossover trials established initial responder rates of 69% to 75% in treatment-naïve patients, and it remains the reference standard against which other agents are compared (63, 64). Its principal limitation in long-term use is a substantial adverse effect burden. Dose-dependent central nervous system effects including dizziness, drowsiness, diplopia, and ataxia are common and particularly problematic in elderly patients (65). Hyponatremia, hepatotoxicity, and rare hematological reactions require periodic monitoring. Potent CYP3A4 induction generates clinically relevant drug interactions that complicate management in patients with comorbid conditions.

Oxcarbazepine is a keto-analogue that is metabolized without passing through the CYP3A4 pathway, resulting in considerably fewer drug interactions and a more favorable tolerability profile (66). A large real-world retrospective study in 354 patients found initial responder rates of 88.3% with carbamazepine and 90.9% with oxcarbazepine, figures somewhat higher than those reported in placebo-controlled trials, likely reflecting the preferential selection of responsive patients in routine clinical practice and the flexibility to optimize dosing outside a controlled trial protocol. No significant difference in efficacy between the two agents was observed, but the rate of treatment discontinuation due to adverse effects was meaningfully lower with oxcarbazepine (67). Oxcarbazepine is therefore increasingly preferred as a first-line alternative, particularly in patients with polypharmacy or known carbamazepine intolerance. Its principal drug-specific risk is hyponatremia, which warrants periodic serum sodium monitoring.

#### 4.1.2 Adjunctive pharmacological agents

For patients with inadequate pain control at maximally tolerated first-line doses, or those unable to tolerate first-line agents, adjunctive pharmacological agents represent the next step. The evidence base for these options is substantially weaker, resting largely on small trials or retrospective series. Lamotrigine acts through sodium channel blockade with an additional reduction of excitatory glutamate release, giving it mechanistic relevance to both peripheral ectopic discharge and central sensitization (68). A single double-blind placebo-controlled trial in 14 patients refractory to carbamazepine demonstrated meaningful pain reduction with adjunctive lamotrigine at 400 mg per day (69). Baclofen, a GABA-B receptor agonist that reduces presynaptic excitatory neurotransmitter release within the trigeminal brainstem complex, has shown efficacy in a single placebo-controlled crossover trial. It is of particular value in patients with MS-related TN, where its antispastic properties provide additional benefit (70). Both agents carry a Level C evidence rating and are considered second-line options when first-line treatments fail.

Gabapentin and pregabalin act on voltage-gated calcium channel α2δ subunits to reduce neurotransmitter release at presynaptic terminals (71). A meta-analysis comparing gabapentin to carbamazepine reported similar overall efficacy with a potentially lower adverse effect rate, though the methodological quality of included studies was variable (72). Pregabalin showed comparable efficacy to lamotrigine with better patient tolerance in a crossover trial, making it a reasonable adjunctive option. It is particularly relevant for patients with concomitant continuous background pain between paroxysms, which first-line sodium channel blockers may not adequately address. Lacosamide, a third-generation sodium channel blocker enhancing slow inactivation, has recently emerged as an additional adjunctive option (73). A retrospective analysis of 86 patients with refractory TN reported pain relief in 66% of cases with oral lacosamide (73). For acute pain exacerbations, intravenous lacosamide achieved pain relief in 77.8% of episodes with a favorable safety profile, supporting its use as a rescue alternative to phenytoin (74). This flexibility across oral and intravenous routes, combined with a mechanism distinct from carbamazepine, makes lacosamide a promising option warranting further controlled evaluation. For patients who fail to achieve acceptable pain control with optimized pharmacological management, the clinical indication for trigger zone-targeted local interventions becomes appropriate.

### 4.2 Trigger zone-targeted local interventions

#### 4.2.1 Local nerve blockade

Local anesthetic agents are injected in proximity to the peripheral trigeminal branch innervating the trigger zone territory (75). They reversibly block voltage-gated sodium channels in the nerve membrane, temporarily abolishing both the trigger response and the resulting pain radiation. The anatomical targets are defined by the location of the patient’s trigger zone, with the supraorbital foramen corresponding to V1-associated zones, the infraorbital foramen to V2 zones in the nasal and upper lip region, and the mental foramen to V3 zones over the chin and lower lip (76).

Beyond its therapeutic role, nerve blockade serves a valuable diagnostic function (77). Significant pain reduction following injection of the targeted branch provides pharmacological confirmation that the branch in question is responsible for generating the patient’s attacks. This diagnostic value is particularly useful when trigger zone location does not clearly correspond to a single division. The introduction of ultrasound guidance has substantially improved precision and safety, enabling real-time visualization of the target nerve and surrounding vascular structures. Accuracy rates of up to 97% have been reported for image-guided supraorbital, infraorbital, and mental nerve blocks compared to landmark-based techniques (78). The primary limitation is the transient nature of analgesia when standard local anesthetics are used, necessitating repeat injections to maintain benefit.

#### 4.2.2 Botulinum toxin type a injections

Botulinum toxin type A has emerged as one of the most clinically promising trigger zone-targeted interventions for TN (79). Its analgesic mechanism is multifaceted and directly relevant to the pathophysiological processes established in the preceding discussion of peripheral and central mechanisms. At the presynaptic terminal, BTX-A cleaves SNAP-25, blocking calcium-dependent vesicular release of substance P, glutamate, and calcitonin gene-related peptide from sensitized trigeminal afferents. It also reduces TRPV1 surface expression at peripheral terminals, contributing to a reduction in peripheral sensitization (80). Emerging evidence suggests that BTX-A undergoes retrograde axonal transport after peripheral injection. This may exert modulatory effects on central sensitization pathways, accounting for its analgesic duration beyond what purely peripheral action would predict.

A meta-analysis by Wei and colleagues pooled 10 randomized controlled trials comprising 391 participants. BTX-A was found superior to placebo for pain intensity reduction at one, two, and 3 months post-injection (81). A separate meta-analysis focusing on four placebo-controlled trials found a responder risk ratio of 2.87 versus placebo (95% CI: 1.76 to 4.69), meaning patients receiving BTX-A were nearly three times more likely to achieve meaningful pain reduction (82). The most common injection approach targets the cutaneous and mucosal territory of the affected trigeminal division with subcutaneous or intradermal injections at 5 units per site. Additional units are directed specifically at clinically identified trigger zone locations. Total doses typically range from 25 to 75 units per session. Adverse effects are predominantly mild and transient, including facial asymmetry and local bruising, and all self-resolve within 1 to 2 weeks. This favorable safety profile makes BTX-A particularly suited to elderly patients with comorbid conditions that preclude aggressive pharmacotherapy or surgical intervention. The 2019 European Academy of Neurology guideline formally acknowledged BTX-A as a weak add-on or alternative therapy for patients with inadequate response to pharmacotherapy (83).

#### 4.2.3 Minimally invasive ablative techniques

When both pharmacotherapy and the less invasive approaches described above fail to achieve adequate pain control, minimally invasive ablative techniques provide an additional tier of intervention (84). Open surgical procedures are considered only thereafter. Percutaneous radiofrequency thermocoagulation is the most extensively studied technique in this context, delivering controlled thermal energy through a needle electrode to selectively disrupt small-diameter nociceptive fibers while attempting to preserve larger-diameter tactile fibers (85). The lesion is created at the Gasserian ganglion or at more distal peripheral branches corresponding to the patient’s trigger zone distribution, under image guidance using fluoroscopy, CT, or increasingly ultrasound-assisted techniques.

Across published retrospective case series and single-arm cohorts, immediate pain relief is achieved in approximately 90% to 100% of patients across published series, making it among the most reliably effective short-term interventions available, though high-quality randomized comparative data remain scarce. The principal limitation is pain recurrence over time, with rates of approximately 29% to 43% reported at 3 to 4 years of follow-up. Repeat thermocoagulation has consistently been shown to restore adequate pain control in the majority of those who experience recurrence. The most common adverse effect is facial hypoesthesia in the treated territory, which is generally mild and well tolerated. Serious complications including troublesome dysesthesia, masticatory weakness, and corneal anesthesia are rare but require careful patient selection and precise lesion targeting. The growing availability of advanced image guidance, including real-time CT fusion with ultrasound, has made it increasingly feasible to target individual peripheral branches corresponding to the patient’s specific trigger zone territory. This improves selectivity while reducing unnecessary involvement of unaffected divisions.

### 4.3 Integrated treatment strategies

The systemic and local approaches described above are not competing alternatives but mechanistically complementary pathways that target different levels of the peripheral-central sensitization continuum. Systemic sodium channel blockers reduce global neuronal excitability at both peripheral and central levels. Local interventions, by contrast, suppress the abnormal afferent input from the trigger zone that sustains central sensitization. Combining these modalities therefore addresses the pathophysiological process from both ends simultaneously.

The clearest controlled evidence for combined therapy comes from a randomized study comparing oral carbamazepine alone against carbamazepine combined with ropivacaine nerve blockade at trigger zone sites (86). The combination group achieved significantly greater pain reduction while requiring lower carbamazepine doses. This suggests that local trigger zone intervention can reduce the systemic drug burden needed to achieve equivalent or superior pain relief. The practical benefit is a potential reduction in the dose-dependent adverse effects that most commonly limit long-term pharmacotherapy.

In clinical practice, the sequence and combination of therapies should be guided by disease stage, trigger zone characteristics, treatment tolerability, and the patient’s overall medical profile. For newly diagnosed patients, systemic pharmacotherapy is the appropriate first step given its established efficacy and non-invasive nature. Trigger zone-targeted interventions are added when pharmacotherapy provides inadequate control, produces intolerable adverse effects, or when bridging analgesia is required during medication titration. It is necessary to contextualize trigger zone-targeted therapies within the full treatment framework of trigeminal neuralgia. Microvascular decompression serves as the primary surgical choice for classical TN with confirmed neurovascular compression, as it eliminates compressive lesions to achieve long-term drug-free pain relief. However, persistent trigger zones and paroxysmal pain are observed in a subset of patients despite successful decompression, implying peripheral and central sensitization can sustain hypersensitivity independent of vascular compression. Trigger zone-directed local interventions and ablative techniques are thus indicated for patients unfit for or refusing craniotomy, those with residual trigger zone-related pain postoperatively, and patients with idiopathic TN without operable neurovascular compression. Patients with a single, anatomically stable trigger zone in a well-defined peripheral nerve territory are the most favorable candidates for local intervention, given the predictability of the target. Those with multiple or shifting trigger zones present a more complex challenge. This group is often best managed by maintaining low-dose systemic therapy for background neural stabilization while directing local interventions at the most clinically dominant trigger zone. Elderly patients and those with comorbid conditions that limit pharmacological options may benefit from earlier introduction of BTX-A given its minimal systemic risk. Long-term management requires dynamic reassessment. Trigger zone characteristics evolve over the disease course, and the relative contributions of peripheral and central mechanisms to an individual patient’s pain may shift over time. High-quality controlled trials that head-to-head compare different therapeutic sequencing strategies are currently lacking. Large-scale controlled studies focusing on trigger zone-targeted therapies are warranted in the future to consolidate the evidence base supporting this clinical management framework.

## 5 Controversies and future research directions

### 5.1 Lack of standardized objective assessment criteria

The identification of trigger zones in clinical practice currently rests almost entirely on patient-reported symptoms and clinician-administered bedside stimulation (87). While this approach captures the essential phenomenology, it introduces substantial variability across clinical encounters and research settings. Reported trigger zone prevalence in published TN cohorts ranges from as low as 36% to as high as 97% (10, 11), a discrepancy that almost certainly reflects methodological inconsistency rather than true biological variation. No validated instrument currently exists for documenting trigger zone location, surface area, activation threshold, or temporal evolution in a reproducible and transferable manner. This gap impairs diagnostic consistency, prevents meaningful longitudinal tracking of trigger zone change within individual patients, and limits the ability to correlate trigger zone properties with treatment outcomes.

Promising directions for addressing this gap include three-dimensional facial sensory mapping and standardized quantitative sensory testing protocols applied specifically to trigger zone regions (88). High-resolution ultrasound assessment of the soft tissue environment overlying identified zones represents a further complementary approach. Future research should prioritize the development and validation of a multimodal trigger zone assessment battery combining these approaches, followed by its prospective application in longitudinal cohorts. Establishing such a framework would also support clearer differentiation of TN trigger zones from myofascial trigger points and migraine-related trigger sites. This would reduce the terminological ambiguity that currently complicates both clinical diagnosis and research design.

### 5.2 Insufficient comparative evidence across treatment strategies

The therapeutic landscape for TN is characterized by a notable mismatch between the volume of published clinical experience and the quality of evidence supporting individual treatment choices (89). Carbamazepine and oxcarbazepine retain their first-line status on the basis of older trials whose designs would not meet contemporary methodological standards. For adjunctive agents and trigger zone-targeted interventions, the evidence base consists predominantly of small single-arm studies, retrospective series, and trials with short follow-up periods and heterogeneous outcome measures. Many interventional studies recruit fewer than 50 participants, and inconsistent injection protocols, operative procedures and assessment standards across medical centers further contribute to cross-trial heterogeneity. Variations in research design may introduce mild risks of selection, performance and publication bias, which partially limit the broad applicability of pooled clinical observations. Most critically, no adequately powered head-to-head randomized controlled trial has directly compared different trigger zone-targeted interventions against one another. No trial has evaluated a combined pharmacological and local intervention strategy against optimized monotherapy either. Clinicians therefore currently make treatment sequencing decisions on the basis of mechanistic rationale and clinical experience rather than direct comparative efficacy data.

Future research priorities should include multicenter randomized trials comparing trigger zone-targeted botulinum toxin type A injection against peripheral nerve blockade in patients with inadequate response to first-line pharmacotherapy (90). Equally important are trials evaluating whether combined systemic and local intervention strategies reduce time to adequate pain control or cumulative drug exposure relative to pharmacotherapy alone. Standardization of outcome domains across trials is an essential prerequisite for meaningful cross-trial synthesis. Domains that require agreement include pain intensity, attack frequency, trigger zone activation threshold, quality of life, and adverse effect burden. Patient stratification by trigger zone distribution, disease duration, and TN subtype should be incorporated into trial designs from the outset, given growing recognition that these variables influence treatment response. Expanding the evidence base along these lines represents the most direct route toward individualized, mechanism-guided management of TN. Consequently, it is difficult to evaluate the completeness of literature coverage or quantify the potential selection bias inherent to this narrative review design.

## 6 Conclusion

Trigger zones constitute the hallmark clinical feature of trigeminal neuralgia and key to its diagnosis and management. Cumulative evidence shows trigger zone hyperexcitability arises from three interrelated processes: voltage-gated sodium channel dysregulation, mechanical sensitization induced by facial and masticatory muscle activity, and trigeminal brainstem central sensitization, consistent with neuroimaging showing supraspinal overactivation upon innocuous trigger zone stimulation. Evidence strength differs markedly across these pathways. The clinical value of trigger zones, sodium channel blocker efficacy, and root entry zone demyelination are well validated, while specific sodium channel subtypes, muscle-nerve crosstalk, and their unifying feedback loop remain unconfirmed hypotheses requiring human tissue verification. These pathways interact bidirectionally: peripheral ectopic discharges maintain central sensitization, which further reduces pain trigger thresholds. This trigger zone-centered analytical framework is the core novelty of the present review. Rather than treating trigger zones as an independent lesion, we frame them as a clinically valuable intermediate phenotype linking peripheral nerve damage and central sensitization. Unlike general TN reviews, this phenotype-focused perspective facilitates targeted, localized interventions. Clinically, trigger zones guide branch identification and personalized drug and interventional treatment decisions. Systemic sodium channel blockers serve as first-line therapy, while peripheral nerve blocks, botulinum toxin injections and minimally invasive ablative techniques provide synergistic adjuvant effects. Future research should develop standardized trigger zone evaluation tools and large comparative trials to advance precision, individualized trigeminal neuralgia care.
